# Complete remission of ALK-negative plasma cell granuloma (inflammatory myofibroblastic tumor) of the lung induced by celecoxib: A case report and review of the literature

**DOI:** 10.3892/ol.2013.1260

**Published:** 2013-03-15

**Authors:** CINDERELLA CHAVEZ, MARK A. HOFFMAN

**Affiliations:** Division of Hematology-Oncology, Department of Medicine, Long-Island Jewish Medical Center, North Shore-LIJ Health System, New Hyde Park, NY 11040, USA

**Keywords:** inflammatory myofibroblastic tumor, non-steroidal anti-inflammatory agents, cyclooxygenase-2 inhibitors

## Abstract

We report a case in which a 52-year-old female developed a multifocal inflammatory myofibroblastic tumor (IMT) of the lung. The tumor did not overexpress the anaplastic lymphoma kinase (ALK) protein, indicating a lack of ALK rearrangement. The patient required two wedge resections in 15 months due to recurrent disease. Recurrence after the second surgery was treated with corticosteroids, which only led to a transient response (6 months). Introduction of celecoxib, a cyclooxygenase-2 inhibitor, induced a complete remission in the patient. Maintenance on celecoxib further led to a progression-free survival of 34 months. A literature review retrieved a total of eight case reports, comprising ten patients, of IMT of various anatomical sites successfully treated with non-steroidal anti-inflammatory agent (NSAID) therapy. Nine of the ten patients achieved durable complete remission. Remission occurred rapidly and persisted even after termination of NSAID therapy. Although such a successful outcome may only be achieved rarely, a trial of an NSAID should be considered in any patient in whom complete resection is not an option. Our case also demonstrates that NSAID therapy may be successful in a non-ALK rearranged tumor in which ALK inhibition is not an option.

## Introduction

Inflammatory myofibroblastic tumor (IMT) is a rare disease entity that has been reported to occur in multiple anatomical locations, including the lung, bladder, spleen, breast, pancreas, liver, colon, prostate, peripheral nerves, soft tissue and orbit (1). IMT has thus far been most commonly referred to in the literature as ‘inflammatory pseudotumor’, ‘plasma cell granuloma’, ‘pseudosarcoma’ or ‘fibromyxoid lesion’.

Histologically, these lesions may exhibit a heterogeneous appearance, thus accounting for the various terms used to describe IMTs alluded to above. All IMTs are characterized by variable cellular spindle cell proliferation with a compact myxoid stromal pattern and a variable inflammatory infiltrate, usually comprised of plasma cells, lymphocytes, eosinophils and neutrophils. The spindle cells possess the morphological appearance of myofibroblasts. Immunohistochemistry of the spindle cells reveals reactivity for smooth muscle actin and desmin, and ultrastructural studies reveal a predominance of myofibroblasts and a smaller fibroblastic component (1).

IMTs may occur in any age group, however, they are observed most commonly in children and adolescents. The most prevalent anatomical sites for IMTs to occur include lung, abdomino-pelvic and retroperitoneal areas, although any site may be involved. Patients may present with non-specific symptoms associated with the site of the mass, including cough and abdominal pain. Constitutional symptoms are present in 15–30% of cases and laboratory evaluation may reveal microcytic anemia, an elevated ESR, thrombocytosis and/or polyclonal hypergammaglobulinemia (1). In some cases, the mass may be detected after an extensive work-up of fever of unknown origin. These systemic symptoms frequently resolve following surgical excision and tumor recurrence may be marked by a return of clinical and laboratory abnormalities.

IMTs are classified as tumors with an intermediate biological potential, in that local recurrences may occur and there is a rare possibility of distant metastasis (2,3).

Previously, insight into the pathogenesis of at least some cases of IMT has been illuminated by the finding of rearrangements of the anaplastic lymphoma kinase (ALK) gene on chromosome 2p23 in 50% of IMT cases. Multiple fusion partners have been identified. ALK expression by immunohistochemistry reliably predicts a rearrangement by FISH or PCR (1).

Complete surgical resection, whenever feasible, is the treatment of choice for IMT (4,5). Ill-circumscribed tumors, particularly in the abdomino-pelvic area, may be difficult to completely resect and local recurrences are not uncommon. Local recurrences are usually managed with re-excision if possible and the vast majority of patients with local recurrence are free of disease with long-term follow-up (4,5).

For patients who are unable to have complete resections, including in the case of multiple lesions or disease in an area where resection would be anatomically difficult, other modalities have been employed. Corticosteroid monotherapy may result in rapid resolution of the disease and sustained remission (6,7). Non-steroidal anti-inflammatory agents (NSAIDs) as solitary therapy may be extremely efficacious (8) and are the subject of this study. Radiation alone may induce enduring remission (9). Anecdotal response to chemotherapy has also been reported (10).

Most recently, a case has been published in which crizotinib, an inhibitor of ALK kinase, induced a partial remission in a patient with an IMT characterized by a RANB2-ALK fusion gene (11). A patient in the same study with an ALK-negative IMT did not respond to crizotinib, supporting ALK inhibition as the basis of the therapeutic effect (11).

We report our experience in the successful treatment of an ALK-negative IMT of the lung, pre-treated with two surgeries and corticosteroid therapy, with a prolonged course of celecoxib and have comprehensively reviewed the literature on NSAID therapy of IMTs. Informed consent was obtained from the patient.

## Case report

### Clinical presentation and diagnosis

A 52-year-old woman was observed to have a pulmonary nodule in the left lower lobe on a chest X-ray, performed as part of preoperative testing for a dilatation and curettage. Computed tomography (CT) scans of the chest performed subsequently showed nodular opacities in the bilateral lower lobes. The patient was managed conservatively and a repeat CT scan performed 2 months thereafter showed almost complete resolution of the nodule in the left lower lobe and complete resolution of the nodule in the right lower lobe. At the time, these findings were thought to represent the resolution of inflammatory disease of the lung.

**Figure 1:**
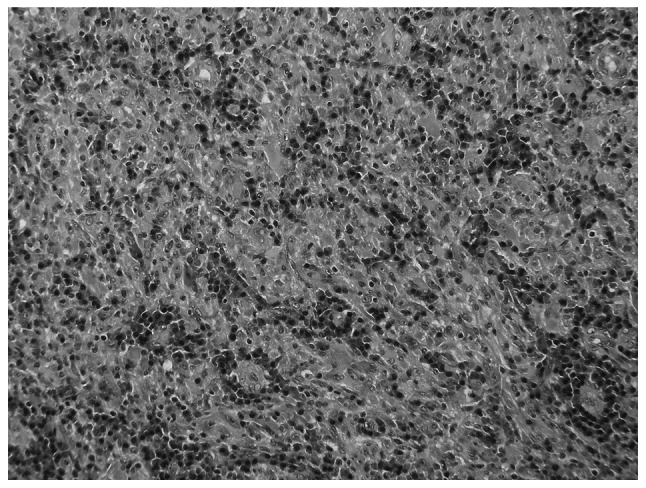
Hematoxylin and eosin. Lung wedge resection revealing the typical histology of an IMT. The image depicts an admixture of mesenchymal cells and plasma cells. IMT, inflammatory myofibroblastic tumor.

In October 2007, viral-type respiratory symptoms led to a CT scan of the chest which revealed a left lower lobe lung nodule. PET/CT scans in November 2007 revealed a 2.3×1.9-cm irregularly shaped nodule in the left lower lobe with minimal uptake (SUV 3.4) as well as a separate 7-mm non-avid nodule. Follow-up chest CT scans in December 2007 revealed that the left lower lobe nodule resolved into two separate nodules, in aggregates larger than the previous. Multiple new pulmonary nodules were observed in both lungs. A wedge resection of the left lower lobe in January 2008 revealed a mass composed of plasma cells admixed with lymphocytes, histiocytes and mesenchymal cells (Fig. 1). Review at the Pathology Department of the National Institutes of Health (Bethesda, MD, USA) confirmed the diagnosis of plasma cell granuloma. We have since performed ALK immunohistochemistry on this specimen, which identified no positivity.

### Treatment and clinical course

**Figure 2:**
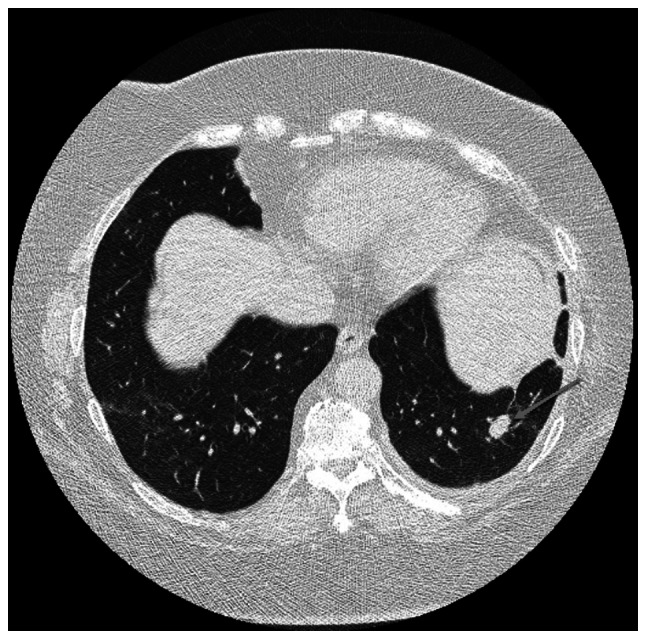
Left lower lobe nodule prior to celecoxib therapy.

**Figure 3:**
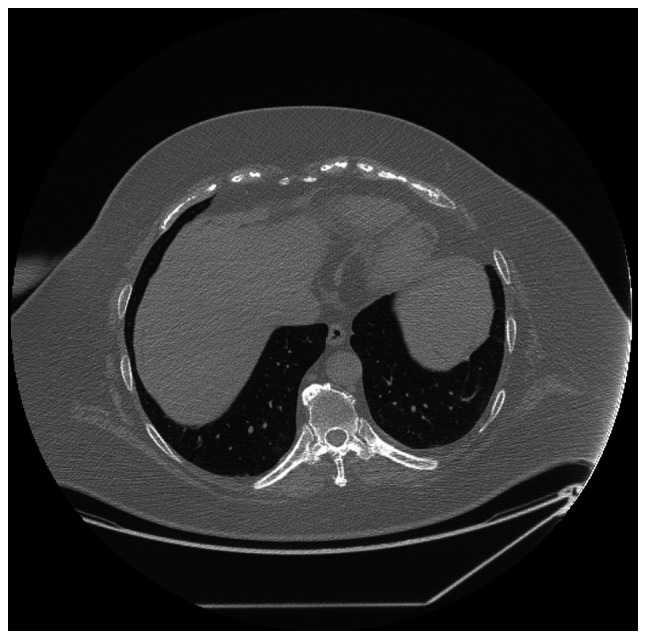
Complete resolution of the left lower lobe nodule following celecoxib therapy.

Follow-up CT scans of the chest were performed thereafter. In March 2009, there was progression of disease with new nodules compared with an assessment performed in December 2008. In April 2009, repeat CT showed increases in size of the left upper lobe and left lingular nodules. The patient underwent surgical resection of the left lingular mass in May 2009, again revealing plasma cell granuloma. Repeat scans performed one month after the second surgery demonstrated progression with multiple nodules involving bilateral lung fields. This prompted treatment with prednisone, initiated in June 2009 at 40 mg/day, which was reduced by October 2009. CT scans in September had revealed resolution of the nodules, however, in December 2009, a follow-up scan highlighted the recurrence of multiple lung nodules involving bilateral lung fields (Fig. 2). In January 2010, the patient was administered celecoxib 200 mg PO BID. By March 2010, CT scans showed improvement with resolution of a nodule and a reduction in size of others, with subsequent scans revealing continuing response to therapy. By February 2012, CT scans demonstrated resolution of all lung nodules (Fig. 3). The patient’s most recent CT scan in August 2012 revealed no progression of disease, 32 months after starting celecoxib. The patient has been reduced to 200 mg every other day.

### Literature review search strategy

A literature search was performed in MEDLINE and EMBASE to identify all published studies using the search terms ‘inflammatory myoblastic tumor’, ‘plasma cell granuloma’, ‘inflammatory pseudotumor’, ‘NSAIDs’, ‘anti-inflammatory agents, nonsteroidal’ and ‘cyclooxgenase 2 inhibitors’. References for all retrieved studies were also reviewed to ensure that no studies had been missed in the primary search.

### Summary of retrieved studies

Altogether, there have been a total of eight previous studies of IMTs managed successfully with NSAID monotherapy, comprising a total of ten patients (Table I). In the first reported case, Hakozaki *et al*(8) treated a female with a 2.5×2.5-cm tumor in the left lobe of the liver. The biopsy revealed ‘edematous granulation tissue with capillaries and fibroblasts’. The patient had experienced abdominal pain for 6 months; one month of non-steroidal therapy induced complete remission (CR) of the mass and resolution of the abdominal discomfort. Su *et al*(12) reported on two patients with intra-abdominal masses. Tissue pathology was not described other than being consistent with an inflammatory pseudotumor (IPT). NSAID monotherapy induced CR after 2 months in one patient and after 4 months in the other patient. In both cases, NSAID therapy was discontinued at the time of achieving CR and the patients subsequently remained in remission. The case report of Chan *et al*(13) is the only case of NSAID therapy of pulmonary IMT other than our own case. The reported patient had an unresectable right lung mass, was febrile and toxic-appearing, with laboratory results indicative of acute inflammation with thrombocytosis, elevated ESR and elevated CRP. Histology revealed fibrous tissue, areas of necrosis and extensive lymphocyte and plasma cell infiltration. Rofecoxib therapy caused marked symptomatic improvement within 72 h; inflammatory parameters normalized and the patient achieved CR after 8 months of therapy. Przkora *et al*(14) published two cases of intra-abdominal IMT. Both patients received a short initial period of steroid treatment along with an NSAID, which was subsequently continued as monotherapy. The response trajectory is not described in either patient. One patient achieved CR after 14 months and the other had stable disease after 12 months. Both patients were maintained on NSAIDs at the time of publication. Colakoglu *et al*(15) reported on a case of a female with a solitary liver mass. NSAID therapy induced CR by 40 days. Vassiliadis *et al*(16) also reported on a patient with a solitary liver mass. In this case, the patient had fever, leucocytosis thrombocytosis and an acute phase reaction serology, i.e., the same clinical presentation as the case of Chan *et al*(13). Naproxen induced a prompt reduction in temperature and was continued for one month. Off therapy, the mass continued to regress and the patient achieved CR. Mattei and Barnaby (17) reported a case in which ketorolac induced a significant response in a large retroperitoneal mass; the patient required surgery within 24 h due to rapid regression of the tumor resulting in a perforated duodenum. Finally, Colangelo *et al*(18) reported a case of a pancreatic head IMT in which ibuprofen induced CR after 6 months. Four years after ceasing drug administration, the patient remained in an unmaintained remission.

Thus, of the ten cases of NSAID therapy reported in the literature, nine were in CR and one had stable disease at the time of publication. The marked paucity of studies in the literature search is noteworthy, and likely reflects a publication bias, in that a study of unsuccessful use of NSAID therapy would not have been submitted for publication.

From these studies, it is apparent that i) responses are not site-specific; ii) responses occur extremely soon after the onset of treatment, indeed as early as 24 h; iii) CRs to NSAID therapy may be extremely durable; iv) remission persists after termination of NSAID therapy.

## Discussion

The induction of durable CR of IMTs with NSAID therapy, albeit a likely rare phenomenon given the scarcity of published studies, prompts speculation as to the mechanism of antitumor activity in this setting.

Cyclooxygenase (COX) is the key enzyme involved in the synthesis of important biological modulators called prostanoids, a collective term for prostaglandins and thromboxanes. Prostanoids are involved in multiple physiological processes, including vasomotility, platelet aggregation, gastrointestinal mucosal integrity, immunomodulation and regulation of cell growth and differentiation (19). There are two major isoforms of COX; COX-1 which is constitutively expressed in all normal tissues and COX-2, which is an enzyme that is inducible by inflammatory and mitogenic signals.

COX-2 products have been demonstrated to be important in cancer development, progression and metastasis (19,20). Prostaglandin E2 (PGE2 ) is particularly significant in mediating tumor progression. Overexpression of COX-2 in tumors leads to progression via multiple mechanisms, including increasing angiogenesis, decreasing apoptosis and increasing invasiveness. PGE2 initiates a downstream growth signaling cascade involving the epidermal growth factor receptor, nuclear receptor ras-mitogen-activated protein kinase pathways and upregulating invasiveness by activating matrix metalloproteinases.

Numerous types of cancer have been demonstrated to over-express COX-2 and overexpression often correlates with more aggressive behaviour (20). Given the importance of COX-2 in enhancing cell growth, COX-2 inhibitors have been employed in a number of clinical settings (19,20). The most notable data have been in the area of chemoprevention, particularly in the reduction of the development of colonic polyps. Despite a large body of work demonstrating a significant antitumor effect in preclinical models, results have been disappointing in the advanced cancer setting thus far.

Applebaum *et al* studied the expression of COX-2, VEGF and ALK in 11 cases of IMT (21). Using a grading system of 1+ to 3+, with 3+ representing the greatest staining intensity, one tumor was graded as 3+, seven as 2+ and one as 1+. VEGF was strongly expressed in these tumors, suggesting an association of COX-2 with increased angiogenesis. On the basis of these data, the authors postulated that COX-2 inhibition may be an effective therapy for IMT. IMT additionally likely includes a spectrum of disease and based on anecdotal data, abdominal tumors may respond more favorably than thoracic tumors to COX inhibition (21).

NSAID therapy may induce enduring CR in untreated or pretreated IMT, as evidenced by our case and the abovementioned studies. Our patient had recurrent disease after surgery, suggesting a more aggressive biology, and corticosteroid therapy only achieved a transient response (6 months). Significantly, our case also demonstrates that NSAID therapy may induce CR in ALK-negative IMTs where ALK inhibition is not a therapeutic option. Excellent response to an NSAID is likely a rare event, however, given the fact that responses may be marked and persist after ceasing therapy, a trial of an NSAID should be considered in any patient with an unresectable or recurrent IMT.

## Figures and Tables

**Table I:** Published studies of NSAID therapy of IMT.

Author (Ref.)	Age (years)	Gender	Primary site	Prior treatment	Therapy (duration)	Result (duration)
Present case	52	F	Lung	Two surgeries Corticosteroids	Celecoxib (32 months)	CR (32 months)
Colangelo *et al*(18)	4	M	Pancreas	None	Ibuprofen (6 months)	CR (4 years)
Mattei *et al*(17)	13	M	Duodenum	None	Ketorolac (24 h)	CR (NS)
Vassiliadis *et al*(16)	16	M	Liver	None	Naproxen (1 month)	CR (1 year)
Colakoglu *et al*(15)	62	F	Liver	None	NSAID (1 month)	CR (1 year)
Przkora *et al*(14)	63	F	Mesentery	None	Diclofenac (ongoing)	CR (14 months)
	22	M	Retroperitoneum	None	Ibuprofen (ongoing)	SD (1 year)
Chan *et al*(13)	7	F	Lung	None	Rofecoxib (8 months)	CR (NS)
Su *et al*(12)	6	F	Pelvis	None	Naproxen (4 months)	CR (2 years)
	14	M	Mesentery	None	Ibuprofen (2 months)	CR (6 months)
Hakozaki *et al*(8)	52	F	Liver	None	Loxoprofen (1 month)	CR (6 months)

CR, complete response; SD, stable disease; NSAID, non-steroidal anti-inflammatory agent; IMT, inflammatory myofibroblastic tumor; F, female; M, male; NS, not stated.
